# Modulare Akutpsychotherapie – Hintergrund, Konzept und Implikationen für die psychiatrisch-psychotherapeutische Weiterbildung: Ein Konsensuspapier der DGPPN

**DOI:** 10.1007/s00115-026-02025-7

**Published:** 2026-09-24

**Authors:** Andreas Bechdolf, Anja Lehmann, Hendrik Müller, Katharina Kubera, Sabine C. Herpertz

**Affiliations:** 1 Klinik für Psychiatrie, Psychotherapie und Psychosomatik mit FRITZ am Urban und Soulspace, Vivantes Klinikum am Urban und Vivantes Klinikum im Friedrichshain, Berlin, Deutschland; 2 Klinik für Psychiatrie und Psychotherapie, CCM, Charité – Universitätsmedizin Berlin, Berlin, Deutschland; 3 Deutsches Zentrum für Psychische Gesundheit (DZPG), Potsdam-Berlin, Deutschland; 4 Klinik und Poliklinik für Psychiatrie und Psychotherapie, Universitätsklinikum Köln, Köln, Deutschland; 5 Klinik für Psychiatrie und Psychotherapie, Universitätsklinikum Heidelberg, Heidelberg, Deutschland; 6 Deutsches Zentrum für Psychische Gesundheit (DZPG), Mannheim-Heidelberg-Ulm, Deutschland

**Keywords:** Akutpsychiatrie, Psychotherapie, Modulare Psychotherapie, Transdiagnostische Behandlung, Weiterbildung, Acute psychiatry, Psychotherapy, Modular psychotherapy, Transdiagnostic treatment, Education, medical, graduate

## Abstract

**Hintergrund:**

Die Anwendung störungsspezifischer Psychotherapie setzt häufig ein Mindestmaß an gemeinsamem Behandlungsauftrag, Zielklärung und therapeutischem Arbeitsbündnis voraus. In der akutpsychiatrischen Behandlung liegen diese Voraussetzungen häufig nicht vor. Diagnosen sind unsicher; gemeinsame Behandlungsziele und ein Arbeitsbündnis müssen oft erst entwickelt werden.

**Ziel der Arbeit:**

Die modulare Akutpsychotherapie (MAPT) wird als integratives psychotherapeutisches Konzept für die besonderen Anforderungen der psychiatrischen Akutbehandlung vorgestellt, und ihre Implikationen für die psychiatrisch-psychotherapeutische Weiterbildung werden diskutiert.

**Material und Methoden:**

MAPT integriert unterschiedliche Ansätze in ein transdiagnostisches, interprofessionelles und modulares Entscheidungs- und Interventionsmodell.

**Ergebnisse:**

Drei dynamische Behandlungsfokusse – Eigengefährdung, Fremdgefährdung und Gefährdung der Therapie – strukturieren die Priorisierung. Vier Module – Risikoreduktion, Zugang zum subjektiven Erleben, Motivationsförderung und die Herstellung eines gemeinsamen Problemverständnisses – unterstützen die Auswahl konkreter Interventionen. Die Beziehungsgestaltung bildet die übergeordnete therapeutische Grundlage. Fokusauswahl und Interventionen werden fortlaufend evaluiert und angepasst.

**Diskussion:**

MAPT bietet ein Entscheidungs- und Behandlungskonzept für die Psychotherapie in der psychiatrischen Akutbehandlung. Psychotherapie wird dabei nicht als nachgelagerter Bestandteil, sondern als therapeutische Perspektive ab der Aufnahme verstanden. Als gemeinsame Denk- und Handlungssystematik kann das Modell zugleich psychotherapeutisches Lernen, interprofessionelle Zusammenarbeit und klinische Supervision strukturieren.

## Abstract (en)

**Background:**

The application of disorder-specific psychotherapy often requires a minimum level of joint treatment, clarification of goals and a therapeutic alliance. In acute psychiatric treatment these prerequisites are not always initially present: admissions can be unplanned or involuntary, diagnostic classification can be uncertain and shared goals and a viable therapeutic alliance first need to be established.

**Objective:**

The modular acute psychotherapy (MAPT) is presented as an integrative psychotherapeutic concept for the specific requirements of acute psychiatric treatment and its implications for psychiatric and psychotherapeutic training are discussed.

**Material and methods:**

The MAPT integrates concepts from different psychotherapeutic approaches and psychiatric care models into a transdiagnostic interprofessional and modular framework for therapeutic decision-making and interventions.

**Results:**

The MAPT distinguishes three dynamic treatment foci: risk of self-harm, risk of harm to others and threats to the therapeutic process. The selection of defined interventions is supported by four modules: risk reduction, access to subjective experience, motivation enhancement and the development of a shared understanding of the problem. Therapeutic relationship building provides the overarching foundation. Clinicians continuously evaluate and adapt the treatment focus and interventions.

**Conclusion:**

The MAPT provides a framework for decision and treatment concepts for psychotherapy in acute psychiatric treatment. In this respect psychotherapy is not understood as a downstream component but as a therapeutic perspective on admission. As a shared framework for therapeutic thinking and action it can also support psychotherapeutic learning, interprofessional collaboration and clinical supervision.

Psychotherapie in der Akutpsychiatrie beginnt häufig unter Bedingungen, unter denen ein gemeinsamer Behandlungsauftrag, diagnostische Klarheit und ein tragfähiges Arbeitsbündnis noch nicht bestehen. Die modulare Akutpsychotherapie (MAPT) bietet einen transdiagnostischen und interprofessionellen Rahmen, um auch in diesen Situationen das psychotherapeutische Handeln systematisch zu planen, durchzuführen und zu evaluieren.

## Psychotherapie in der Akutpsychiatrie

Für schwere psychische Erkrankungen stehen gut untersuchte und in Leitlinien empfohlene Psychotherapiemethoden zur Verfügung [1, 2]. Die klinische Ausgangslage psychiatrischer Akutbehandlung unterscheidet sich jedoch häufig von den Bedingungen geplanter Psychotherapie. Ein großer Anteil der Aufnahmen in pflichtversorgenden psychiatrischen Kliniken erfolgt ungeplant oder notfallmäßig [3]. Betroffene können gegen ihren Willen aufgenommen werden, diagnostische Einordnungen können zunächst unsicher sein, Komorbiditäten sind häufig und ein therapeutisches Arbeitsbündnis besteht häufig noch nicht [4, 5]. Die von Betroffenen formulierten Anliegen und die von professionell Tätigen wahrgenommenen Behandlungsnotwendigkeiten können erheblich voneinander abweichen. In psychiatrischen Akutsituationen zeigt sich ein grundlegendes Problem: Die Beteiligten teilen oft weder dieselbe Situationsdeutung noch dieselben Annahmen über Ursachen oder Lösungen. Patient:innen erleben sich beispielsweise bedroht, unverstanden oder verzweifelt oder haben sich von der konsensuellen Wirklichkeit entfernt, was zu Sichtweisen führt, die für andere nicht unmittelbar nachvollziehbar sind [6]. Gleichzeitig sorgen sich Angehörige um die Sicherheit und die Zukunft der Patient:innen. Behandelnde müssen gleichzeitig Risiken einschätzen, rechtliche Vorgaben beachten und Entscheidungen unter Zeitdruck treffen. Was aus professioneller Perspektive eine notwendige Schutzmaßnahme ist, kann für eine betroffene Person eine Bedrohung darstellen. Erfahrungen dieser Art und deren Bedeutung für Beteiligung, Beziehung und Entscheidungsprozesse sind in qualitativen und systematischen Arbeiten gut beschrieben [4, 5].

Diese Bedingungen sind nicht bloße Hindernisse, die überwunden werden müssen, bevor Psychotherapie beginnen kann. Sie sind vielmehr zentrale Gegenstände psychotherapeutischen Handelns in der Akutpsychiatrie. Die Antwort kann deshalb nicht darin bestehen, mit der Psychotherapie zu warten, bis diagnostische Klarheit, Behandlungsmotivation und ein gemeinsames Krankheitsverständnis vorliegen. Psychotherapeutisches Handeln muss vielmehr auch unter den Bedingungen umgesetzt werden, unter denen psychiatrische Akutbehandlung tatsächlich erfolgt.

Dafür ist eine therapeutische Konzeption erforderlich, die Orientierung ermöglicht, ohne die Komplexität der Situation vorschnell zu reduzieren. Eine solche Konzeption muss unter diagnostischer Unsicherheit funktionieren, unterschiedliche Perspektiven integrieren, Veränderungen der klinischen Situation berücksichtigen und auch dann therapeutische Handlungsmöglichkeiten eröffnen, wenn noch kein gemeinsamer Behandlungsauftrag besteht. Die modulare Akutpsychotherapie (MAPT) wurde mit diesem Ziel entwickelt.

## Grundlagen der modularen Akutpsychotherapie

Modulare Akutpsychotherapie ist keine neue Psychotherapieschule und soll bestehende störungsspezifische Psychotherapieverfahren nicht ersetzen. Sie versucht vielmehr, vorhandenes psychotherapeutisches Wissen für die besonderen Bedingungen der psychiatrischen Akutversorgung nutzbar zu machen. Ziel ist therapeutische Flexibilität, ohne Beliebigkeit. MAPT ist transdiagnostisch, verfahrensübergreifend und interprofessionell konzipiert. Das Konzept schließt an die Entwicklung modularer und personalisierter Psychotherapie an, in der funktional definierte Behandlungselemente flexibel und anhand klinischer Entscheidungsregeln kombiniert werden [7–10].

## Empirischer Stand modularer Psychotherapie

Modularer Psychotherapie gliedert evidenzbasierte Interventionen in klar definierte, kombinierbare Einheiten und passt die Auswahl dieser Module an individuelle klinische Anforderungen an [7]. Für verschiedene Störungsgruppen liegen inzwischen Wirksamkeitsdaten zu modularen und personalisierten Ansätzen vor [9, 10]. Eine aktuelle Übersicht beschreibt modulare Psychotherapie als einen Weg zu stärker personalisierten, mechanismenorientierten Behandlungen und weist zugleich auf offene Fragen der Modulauswahl, Sequenzierung, Adhärenz und Implementierung hin [8]. MAPT überträgt dieses Grundprinzip nicht auf spezifische Diagnosen, sondern auf die besonderen Entscheidungs- und Interventionsanforderungen psychiatrischer Akutsituationen.

## Verhalten im Kontext verstehen

Eine zentrale Annahme von MAPT besteht darin, dass das Verhalten eines Menschen zunächst im Kontext seiner aktuellen Situation, seines subjektiven Erlebens, seiner Erfahrungen und seiner Motive zu verstehen ist. Dies gilt auch für Verhaltensweisen, die im akutpsychiatrischen Alltag als besonders herausfordernd erlebt werden. Die Ablehnung einer Behandlung kann beispielsweise dem Versuch dienen, bedrohte Autonomie wieder herzustellen [11]. Rückzug kann Schutz vor Überforderung bieten [12]. Aggressives Verhalten kann unter anderem mit Angst, Bedrohungserleben oder dem Versuch, Kontrolle zurückzugewinnen, verbunden sein. Ein solches Verständnis bedeutet weder Risiken zu relativieren noch problematisches Verhalten zu akzeptieren. Es erweitert vielmehr die therapeutischen Handlungsmöglichkeiten.

## Motivation als Gegenstand der Behandlung

Behandlungsmotivation wird in MAPT nicht als notwendige Voraussetzung für psychotherapeutische Unterstützung verstanden. Gerade Menschen mit schweren psychischen Erkrankungen teilen zu Beginn einer Akutbehandlung nicht immer die Behandlungsziele des professionellen Systems. Wird vorhandene Veränderungs- oder Behandlungsmotivation zur Voraussetzung psychotherapeutischer Arbeit gemacht, werden damit gerade diejenigen Patient:innen ausgeschlossen, die besonders auf Unterstützung angewiesen sind. Die therapeutische Aufgabe besteht daher initial darin, zu verstehen, welche Anliegen, Motive und Befürchtungen das gegenwärtige Handeln bestimmen und wie trotz unterschiedlicher Perspektiven eine ausreichend tragfähige gemeinsame Arbeitsgrundlage entstehen kann. Konzepte gemeinsamer Entscheidungsfindung unterstützen eine solche partizipative Ausrichtung [13].

## Von der Diagnose zur therapeutischen Aufgabe

Diagnosen ermöglichen fachliche Kommunikation, unterstützen Indikationsentscheidungen und bilden die Grundlage zahlreicher therapeutischer, rechtlicher und organisatorischer Entscheidungen. Für die unmittelbare Frage, was in einer konkreten akutpsychiatrischen Situation therapeutisch zu tun ist, reichen kategoriale Diagnosen jedoch häufig nicht aus. MAPT versteht Diagnostik deshalb nicht als Endpunkt, sondern als Ausgangspunkt therapeutischen Handelns. Diagnostische Informationen, psychopathologischer Befund, biografische Erfahrungen, aktuelle Belastungen und Ressourcen sowie die subjektive Perspektive der betroffenen Person werden zu einem therapeutischen Verständnis der aktuellen Situation zusammengeführt. Dies entspricht den aktuellen Bestrebungen, Psychotherapie stärker transdiagnostisch, transtheoretisch und an Veränderungsprozessen auszurichten [14].

Die leitende Frage verschiebt sich damit von „Welche Erkrankung liegt vor?“ hin zu „Was ist gegenwärtig die wichtigste therapeutische Aufgabe – und was könnte dieser Person helfen, wieder handlungsfähiger zu werden?“

## Modulare Akutpsychotherapie

Daher beginnt MAPT mit einer vorläufigen therapeutischen Fallkonzeption. Auf dieser Grundlage wird der aktuelle Behandlungsfokus priorisiert, geeignete therapeutische Module ausgewählt, Interventionen durchgeführt und deren Wirkung evaluiert.

Diagnostische und klinische Informationen → gemeinsames therapeutisches Verständnis → Priorisierung des Behandlungsfokus → Auswahl therapeutischer Module → konkrete Intervention → Evaluation und Anpassung

**Abb. 1:**
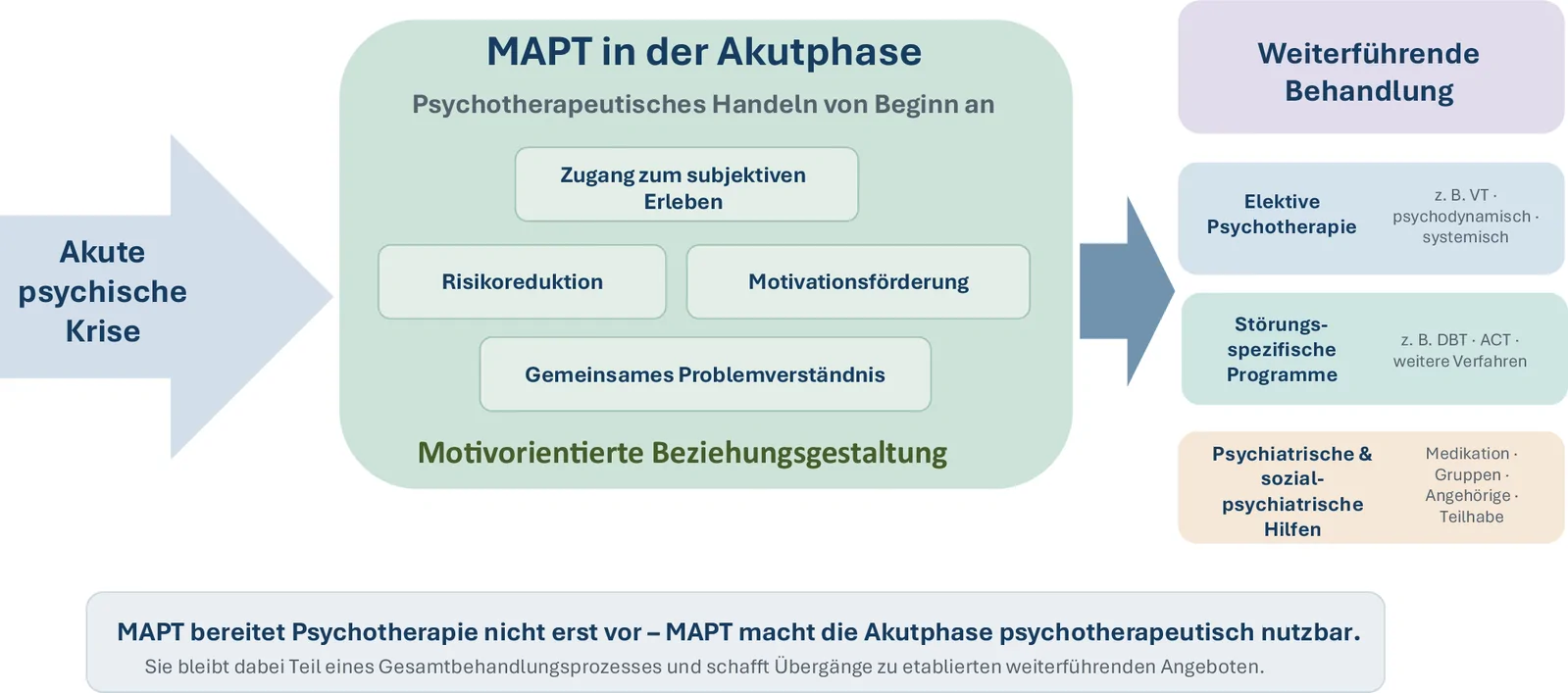
Modulare Akutpsychotherapie (*MAPT*) – Struktur

Modulare Akutpsychotherapie stellt damit ein rekursives therapeutisches Entscheidungs- und Interventionsmodell dar. Abb. 1 zeigt MAPT im Überblick.

## Behandlungsfokus

Akutpsychiatrische Situationen können sich in kurzer Zeit ändern. Ein therapeutisches Modell muss deshalb Prioritäten setzen können, ohne eine statische Behandlungshierarchie festzulegen. MAPT unterscheidet drei Behandlungsfokusse: Eigengefährdung, Fremdgefährdung und Gefährdung der Therapie [15]. Ein Behandlungsfokus bezeichnet den Problembereich, der im gegenwärtigen Behandlungsabschnitt die größte Aufmerksamkeit erfordert und das therapeutische Handeln priorisiert. Die Fokusse sind ausdrücklich nicht Diagnosen zugeordnet. Patient:innen unterschiedlicher diagnostischer Gruppen können mit demselben Fokus behandelt werden; zugleich kann sich der Fokus bei derselben Person im Verlauf rasch verändern.

Bei akuter Suizidalität kann beispielsweise zunächst Eigengefährdung im Vordergrund stehen. Nach ausreichender Distanzierung kann die Aufrechterhaltung der Behandlung oder die drohende Beendigung der Zusammenarbeit zur vorrangigen Aufgabe werden. Umgekehrt kann bei einer zunächst im Vordergrund stehenden Therapiegefährdung eine neu auftretende akute Selbst- oder Fremdgefährdung eine unmittelbare Fokusschiebung erforderlich machen.

## Module

Von den Behandlungsfokussen unterscheidet MAPT die Interventionsebene. Hier stehen vier funktional definierte Hauptmodule zur Verfügung: Risikoreduktion, Zugang zum subjektiven Erleben, Motivationsförderung und Herstellung eines gemeinsamen Problemverständnisses. Beziehungsgestaltung verbindet diese Module zu einer übergreifenden therapeutischen Grundlage. Module sind weder einzelne Interventionen noch verkürzte Psychotherapiemethoden. Sie bezeichnen therapeutische Funktionen, die durch Interventionen unterschiedlicher psychotherapeutischer Herkunft erfüllt werden können [7, 8].

## Risikoreduktion

Das Modul Risikoreduktion dient dazu, akute Gefährdung zu verstehen und so weit zu reduzieren, dass darüber hinausgehende therapeutische Arbeit möglich wird. Risikoeinschätzung und therapeutische Intervention werden dabei nicht als voneinander unabhängige Vorgänge betrachtet. Bereits die Art, wie nach Suizidalität, Gewaltimpulsen oder Kontrollverlust gefragt wird, beeinflusst Beziehung, Offenheit und Kooperationsmöglichkeiten. Ziel ist deshalb nicht ausschließlich die maximale Sicherheit. Sicherheit muss so weit wie möglich gemeinsam hergestellt werden, ohne Autonomie stärker einzuschränken als notwendig.

## Zugang zum subjektiven Erleben

Bevor Veränderung möglich wird, muss zunächst verstanden werden, wie eine Person ihre Situation erlebt. Das Modul dient nicht primär der Herstellung von Krankheitseinsicht. Ziel sind zunächst Orientierung, Validierung, Stabilisierung und Beziehung. Gerade bei psychotischem Erleben, Misstrauen oder hoher emotionaler Anspannung kann der Versuch, vorschnell ein gemeinsames Erklärungsmodell herzustellen, die therapeutische Distanz eher vergrößern. Zugang zum subjektiven Erleben bedeutet, eine Perspektive verstehen zu können, ohne sie vollständig teilen zu müssen.

## Motivationsförderung

Motivationsförderung setzt dort an, wo Ziele von Patient:innen und dem Behandlungsteam nicht oder nur teilweise übereinstimmen. Im Mittelpunkt stehen persönliche Anliegen, Ambivalenzen, Befürchtungen sowie mögliche Veränderungsziele. Ziel ist nicht die schnellstmögliche Zustimmung zu einem vorgegebenen Behandlungsplan. Vielmehr sollen vorhandene Motive sichtbar werden und mögliche Schnittmengen zwischen den Anliegen der betroffenen Person und therapeutisch sinnvollen nächsten Schritten entwickelt werden [13].

## Gemeinsames Problemverständnis

Psychiatrische Krisen werden häufig unterschiedlich erklärt. Patient:innen, Angehörige und verschiedene Mitglieder des Behandlungsteams können dieselbe Situation aus unterschiedlichen Perspektiven beschreiben. Ein gemeinsames Problemverständnis setzt deshalb keine vollständige Übereinstimmung voraus. Es bezeichnet vielmehr eine ausreichend geteilte Arbeitsgrundlage, auf deren Basis die Beteiligten die nächsten Schritte vereinbaren können. Gerade in der Akutpsychiatrie kann ein pragmatischer Konsens wichtiger sein als eine abschließende Einigung über Krankheitsursachen.

## Beziehungsgestaltung als verbindende Grundlage

Beziehungsgestaltung findet nicht neben den Modulen statt. Jede Risikoeinschätzung, jede Motivationsarbeit und jeder Versuch eines gemeinsamen Problemverständnisses vollziehen sich innerhalb einer Beziehung. Die therapeutische Allianz ist über unterschiedliche Psychotherapieverfahren hinweg robust mit dem Behandlungsergebnis assoziiert [16]. Dies gilt auch für Situationen, in denen Grenzen gesetzt, Entscheidungen gegen den aktuellen Willen einer Person getroffen oder Schutzmaßnahmen ergriffen werden müssen. Klinische Entscheidungen haben nicht nur unmittelbare Auswirkungen auf Risiken und Symptome; die Art ihrer Vorbereitung, Begründung, Durchführung und Nachbesprechung beeinflusst zugleich das Vertrauen und die Möglichkeit weiterer Zusammenarbeit. Beziehungsgestaltung ist deshalb keine Intervention, die zu einem bestimmten Zeitpunkt „eingesetzt“ wird, sondern die therapeutische Grundlage des gesamten Prozesses.

## Evaluation

Die Durchführung einer Intervention beendet den therapeutischen Entscheidungsprozess nicht. MAPT sieht eine kontinuierliche Evaluation vor: Hat sich das Risiko verändert? Ist mehr Kontakt möglich? Hat sich die Perspektive der betroffenen Person verändert? Gibt es eine neue gemeinsame Arbeitsgrundlage? Besteht der bisherige Fokus weiterhin? Benötigen wir ein anderes Modul oder eine andere Intervention? MAPT folgt damit einem zyklischen Prozess aus Verstehen – Priorisieren – Handeln – Evaluieren.

## Psychotherapie im akutpsychiatrischen Setting

Das klassische 50-minütige Einzelgespräch ist eine mögliche, aber nicht die einzige Form psychotherapeutischen Handelns in der Akutpsychiatrie. Ein längeres Gespräch kann einen hoch angespannten oder unter psychotischen Symptomen leidenden Menschen überfordern. Umgekehrt können kurze, wiederholte Interventionen durch unterschiedliche Teammitglieder therapeutisch hochrelevant sein. Auch aktuelle fachpolitische Stellungnahmen betonen, dass psychotherapeutische Versorgung in der stationären Psychiatrie nicht auf 50-minütige Einzelpsychotherapie reduziert werden kann, sondern auch kürzere Interventionen, z. B. von 25 min oder kürzer, sowie Gruppenformate einschließt [17].

Aufnahme, Übergabe, Visite, Angehörigengespräch, Stationsregeln, Krisenintervention, Nachbesprechung einer Eskalation und Entlassungsplanung sind Situationen, in denen therapeutische Beziehungen gestaltet, Autonomie ermöglicht oder eingeschränkt, gemeinsame Verständnisse entwickelt und Handlungsfähigkeit gefördert werden können. Dass Veränderungen des Stationsmilieus und der Interaktionen zwischen Mitarbeitenden und Patient:innen Konflikt- und Zwangsereignisse beeinflussen können, zeigt unter anderem eine *Safewards-Studie *[6].

## Didaktik und Weiterbildung

Die psychiatrisch-psychotherapeutische Weiterbildung steht vor einem besonderen Problem. Ärzt:innen in Weiterbildung begegnen häufig bereits zu Beginn ihrer klinischen Tätigkeit Menschen mit schweren psychischen Erkrankungen und komplexen Krisensituationen. Psychotherapeutische Kompetenz lässt sich in einer solchen Situation nicht nur durch theoretisches Wissen erwerben. Therapeutisches Handeln muss im klinischen Alltag beobachtet, unter Supervision erprobt, reflektiert werden [18, 19].

Gerade Berufsanfänger:innen benötigen zunächst Strukturen, die Komplexität reduzieren, ohne starre Handlungsanweisungen vorzugeben. MAPT gibt hier eine wiederkehrende Denkstruktur vor: Situation verstehen → Fokus bestimmen → Modul auswählen → Intervention planen → Wirkung evaluieren.

## Vom regelgeleiteten zum flexiblen Handeln

Berufsanfänger:innen benötigen nachvollziehbare Regeln und Orientierung. Mit wachsender Erfahrung müssen sie zugleich lernen, diese flexibel auf individuelle Situationen anzuwenden. Ein modularer Ansatz bietet hierfür eine geeignete Zwischenebene im klinischen Alltag. Er schreibt nicht für jede klinische Situation eine konkrete Intervention vor, lässt therapeutische Entscheidungen jedoch auch nicht vollständig offen. Die therapeutische Funktion eines Moduls bleibt definiert; die konkrete Intervention kann an Person, Situation, therapeutische Kompetenz und Verlauf angepasst werden. MAPT kann dabei helfen, Flexibilität als Kompetenz zu vermitteln.

## Eine gemeinsame Sprache für das Team

Psychotherapeutische Weiterbildung in der Akutpsychiatrie findet nicht unabhängig vom interprofessionellen Team statt. Patient:innen begegnen im Laufe eines Tages unterschiedlichen Berufsgruppen, die jeweils andere Beobachtungen machen und verschiedene therapeutische Funktionen übernehmen. Eine gemeinsame Sprache über Fokus, Ziele, Module und Evaluation kann unterschiedliche fachliche Perspektiven miteinander verbinden. Für die ärztliche Weiterbildung bedeutet dies auch, psychotherapeutische Kompetenz nicht ausschließlich als Fähigkeit zur Durchführung eigener Einzelpsychotherapie zu verstehen. Dazu gehört ebenso die Fähigkeit, therapeutische Prozesse im multiprofessionellen Team zu erkennen, zu koordinieren und gemeinsam zu reflektieren.

## Implikationen für die Musterweiterbildungsordnung

Die DGPPN hat die Qualität der Facharztweiterbildung Psychiatrie und Psychotherapie zuletzt ausdrücklich als Entwicklungsfeld adressiert und dabei unter anderem Anleitung und Supervision hervorgehoben [20]. In den aktuellen Überlegungen zur Weiterentwicklung der Musterweiterbildungsordnung sollen psychotherapeutische Kurzinterventionen in der psychiatrischen Akutversorgung stärker abgebildet werden. In dem aktuellen Entwurf wurde zur Aufwertung der psychotherapeutischen Kurzinterventionen eine neue Richtzahl von 75 Einheiten à 4 Sitzungen von 25 min Dauer unter Supervision beantragt. Somit umfassen die psychotherapeutischen Kurzinterventionen in der von der DGPPN empfohlenen MWBO neuerdings insgesamt 125 h. Damit wird ein Tätigkeitsfeld sichtbar, das einen erheblichen Anteil an psychiatrisch-psychotherapeutischer Arbeit ausmacht, in bisherigen Weiterbildungslogiken jedoch nur begrenzt als eigenständige Kompetenz beschrieben wurde. Mit MAPT liegt ein Konzept vor, das die Denk- und Handlungssystematik akutpsychiatrischer Kurzinterventionen lehr- und lernbar macht und zugleich interprofessionelle Zusammenarbeit sowie klinische Supervision strukturieren kann.

## Forschungsbedarf und Perspektiven

Modulare Akutpsychotherapie ist gegenwärtig als klinisches und didaktisches Konzept zu verstehen. Ein spezifischer empirischer Wirksamkeitsnachweis für das Gesamtmodell steht aus. Die wissenschaftliche Evaluation sollte deshalb schrittweise erfolgen und zunächst Akzeptanz, Durchführbarkeit und Implementierbarkeit sowie die zuverlässige Anwendung der zentralen Modellkomponenten untersuchen.

## Fazit für die Praxis

- Psychotherapeutisches Handeln sollte in der Akutpsychiatrie mit Beginn der Behandlung einsetzen und weder diagnostische Klarheit noch ein bereits etabliertes Arbeitsbündnis noch eine vorhandene Behandlungsmotivation voraussetzen.
- Modulare Akutpsychotherapie strukturiert komplexe Akutsituationen durch die dynamische Priorisierung der Behandlungsfokusse Eigengefährdung, Fremdgefährdung und Gefährdung der Therapie.
- Die Module Risikoreduktion, Zugang zum subjektiven Erleben, Motivationsförderung und gemeinsames Problemverständnis beschreiben therapeutische Funktionen und ermöglichen eine flexible Auswahl konkreter Interventionen.
- Beziehungsgestaltung und kontinuierliche Evaluation verbinden die einzelnen Schritte des Behandlungsprozesses.
- Modulare Akutpsychotherapie kann zugleich als gemeinsame therapeutische Sprache multiprofessioneller Teams sowie als Struktur für psychotherapeutisches Lernen und Supervision in der multiprofessionellen und ärztlichen Weiterbildung dienen.

## Data Availability

Alle dieser Arbeit zugrunde liegenden Daten können auf begründete Anfrage beim Korrespondenzautor angefordert werden.
